# Detecting anti-forensic deepfakes with identity-aware multi-branch networks

**DOI:** 10.3389/fdata.2025.1720525

**Published:** 2025-12-10

**Authors:** Mingyu Zhu, Jun Long

**Affiliations:** 1 Dundee International Institute, Central South University, Changsha, China; 2 Big Data Institute, Central South University, Changsha, China

**Keywords:** AI-generated content, image processing, multimedia forensics, texture analysis, multi-modal

## Abstract

Deepfake detection systems have achieved impressive accuracy on conventional forged images; however, they remain vulnerable to anti-forensic or adversarial samples deliberately crafted to evade detection. Such samples introduce imperceptible perturbations that conceal forgery artifacts, causing traditional binary classifiers—trained solely on real and forged data—to misclassify them as authentic. In this paper, we address this challenge by proposing a multi-channel feature extraction framework combined with a three-class classification strategy. Specifically, one channel focuses on extracting identity-preserving facial representations to capture inconsistencies in personal identity traits, while additional channels extract complementary spatial and frequency domain features to detect subtle forgery traces. These multi-channel features are fused and fed into a three-class detector capable of distinguishing real, forged, and anti-forensic samples. Experimental results on datasets incorporating adversarial deepfakes demonstrate that our method substantially improves robustness against anti-forensic attacks while maintaining high accuracy on conventional deepfake detection tasks.

## 1 Introduction

In recent years, the rapid advancement of deep generative models has led to the proliferation of highly realistic *deepfakes*—synthetic images and videos generated or manipulated by techniques such as Generative Adversarial Networks (GANs) and diffusion models (Liu et al., 2024b; Lin et al., 2024a). These media forgeries can convincingly mimic facial appearance, expressions, and even voice, posing significant threats to privacy, security, and public trust. The misuse of deepfake technology has been reported in disinformation campaigns, political manipulation, and identity fraud, underscoring the urgent need for reliable deepfake detection systems (Lin et al., 2024b).

Deepfake detection has evolved considerably in the past few years, with methods ranging from handcrafted feature analysis to end-to-end deep neural networks. Early approaches exploited statistical inconsistencies in pixel intensity, color distribution, or compression artifacts, while more recent approaches leverage convolutional neural networks (CNNs), Vision Transformers (ViTs), and multi-modal fusion to automatically learn discriminative forgery patterns from large-scale datasets (Liu et al., 2024a). These methods have demonstrated strong performance in controlled settings, particularly when trained and tested on the same forgery generation method (Chang et al., 2021).

However, most existing detectors operate under the implicit assumption that forgeries exhibit visually detectable or statistically measurable artifacts. While this assumption holds for conventional deepfake samples, it breaks down in the presence of *anti-forensic* (adversarial) manipulations. Anti-forensic techniques (Fan et al., 2023), often derived from adversarial attack paradigms, are designed to deliberately conceal or suppress the traces that detectors rely on (Wang et al., 2022). By introducing imperceptible perturbations to forged content, these methods can drastically reduce the confidence of a detection model, leading to false negatives (Fan et al., 2025; Ding et al., 2024). Alarmingly, such perturbations are often imperceptible to human observers, making manual verification ineffective.

The concept of anti-forensics (Ding et al., 2022b) in multimedia forensics (Ding et al., 2020) predates deepfake technology, originally referring to any technique that intentionally manipulates data to thwart forensic analysis. In the deepfake era, anti-forensic attacks have become more sophisticated and targeted (Ding et al., 2021). Recent studies have shown that even state-of-the-art detection networks can be deceived by relatively small, targeted perturbations generated through optimization-based methods or generative models (Hou et al., 2023). These attacks exploit the inherent vulnerability of neural networks to distribution shifts and adversarial noise (Cao and Gong, 2021).

For instance, an attacker might first generate a manipulated face using a GAN (Fan et al., 2024) or diffusion model, and then apply a crafted perturbation that minimizes the detector's activation on forgery-related features. Without explicit exposure to such samples during training, the detector tends to misinterpret them as authentic, posing a significant security risk in high-stakes applications such as law enforcement, content moderation, and digital identity verification.

In general, Anti-forensic samples refer to fake samples that have been further processed to gain the ability to evade detection, and this processing is typically referred to as an adversarial attack. In contrast, forgery samples are simply fake samples intentionally generated and mixed with real ones to mislead observers, but they do not inherently possess the capability to evade detection. We have explained these terms in the revised manuscript.

On the other hand, defending against anti-forensic attacks is inherently challenging for several reasons:

- **Feature suppression**: adversarial perturbations are optimized to mask the very features that conventional detectors use for classification, rendering feature-based defenses less effective.
- **Data scarcity**: anti-forensic samples are more difficult to collect in large quantities compared to conventional deepfakes, leading to limited training data for robust modeling.
- **Overfitting risk**: models trained with naive augmentation of adversarial samples may overfit to specific perturbation patterns, failing to generalize to unseen attack strategies.
- **Identity consistency exploitation**: many detectors ignore high-level semantic cues such as identity consistency, focusing instead on low-level texture or frequency anomalies, which adversaries can manipulate more easily.

These challenges highlight the need for a more holistic defense strategy that leverages complementary cues beyond low-level artifacts. To address the above challenges, we propose a **multi-channel feature extraction framework** specifically designed to enhance robustness against anti-forensic deepfakes (Ding et al., 2021). Our approach introduces two major innovations:

1. **Identity-preserving channel**: this channel focuses on extracting facial representations that encode identity-consistent features, such as facial geometry, keypoint configuration, and deep identity embeddings. By emphasizing high-level semantic information, this channel captures inconsistencies between the claimed identity and the manipulated content—cues that are more robust to adversarial perturbations targeting low-level features.
2. **Artifact-sensitive channels**: in parallel, we extract complementary features from both the spatial and frequency domains. The spatial domain branch captures local texture irregularities and blending artifacts, while the frequency domain branch highlights abnormal spectral patterns introduced during synthesis and post-processing. These channels retain sensitivity to subtle manipulation traces that may be partially suppressed by anti-forensic perturbations.

The outputs of these channels are fused to form a unified representation, which is then passed to a three-class classifier that distinguishes between *real, conventional forged*, and *anti-forensic forged* samples. This explicit three-class formulation prevents the model from collapsing anti-forensic samples into the “real” class, enabling it to learn decision boundaries that better separate the three categories.

Our method offers several advantages over conventional binary detectors:

- **Enhanced robustness**: by combining identity consistency analysis with multi-domain artifact detection, our model resists attacks that target only one type of cue.
- **Explicit anti-forensic awareness**: the three-class setup forces the detector to learn distinct representations for adversarially perturbed content, improving its ability to flag unseen attacks.
- **Generalization to unseen manipulations**: leveraging complementary channels reduces reliance on any single set of features, enabling better cross-dataset and cross-attack generalization.
- **Compatibility with existing pipelines**: the multi-channel structure can be integrated into existing deepfake detection frameworks with minimal architectural changes, making it practical for deployment.

The key contributions of this work can be summarized as follows:

1. We identify and address the vulnerability of deepfake detectors to anti-forensic (adversarial) manipulations, which are increasingly relevant in real-world scenarios.
2. We propose a novel multi-channel framework that jointly captures identity-preserving facial features and artifact-sensitive spatial/frequency features.
3. We demonstrate through extensive experiments that our approach significantly improves detection performance against anti-forensic attacks while maintaining competitive accuracy on standard deepfake detection benchmarks.

By explicitly modeling the distinctions between conventional and anti-forensic forgeries, our work moves beyond the traditional binary paradigm and provides a more secure and robust deepfake detection strategy. The injected adversarial should be easier to eliminate. In doing so, it not only strengthens defenses against current attack methods but also lays the foundation for countering future, more sophisticated adversarial manipulations.

The remainder of this paper is organized as follows. Section 2 reviews related work on deepfake detection, adversarial attacks, and anti-forensic techniques. Section 3 introduces the proposed multi-channel feature extraction framework. Section 4 details the experimental setup, including datasets, adversarial sample generation, and evaluation metrics. It also presents and analyzes the experimental results, followed by discussions on robustness, generalization, and limitations. Finally, Section 5 concludes the paper and outlines potential directions for future research.

## 2 Related work

### 2.1 Deepfake generation and AI-generated image synthesis

The evolution of deepfake generation techniques can be traced back to early face-swapping algorithms based on 3D morphable models (3DMMs) and traditional computer graphics pipelines. These early approaches, though limited in realism and temporal coherence, laid the foundation for data-driven manipulation. The introduction of deep learning—particularly generative adversarial networks (GANs)—revolutionized the field. Methods such as DeepFake (2017), FaceSwap, and Face2Face leveraged autoencoders and convolutional neural networks to perform identity replacement with increasing visual fidelity. More advanced models like StyleGAN (Karras et al., 2019) and diffusion-based generative models [e.g., DALL·E 2 (Ramesh et al., 2022), Stable Diffusion (Rombach et al., 2022)] expanded capabilities beyond simple face swapping, enabling photorealistic synthesis of arbitrary subjects and scenes. These advances have significantly reduced the technical barrier for forgery creation, allowing even non-expert users to generate convincing manipulated content. At the same time, the rapid growth of AI-generated image technologies has blurred the boundary between creative content generation and malicious forgery, posing serious challenges for digital media authentication.

### 2.2 Deepfake detection techniques

In response to the proliferation of deepfakes, a wide range of detection methods have been proposed. Early works relied on hand-crafted features capturing visual inconsistencies, such as mismatched facial landmarks (Li and Lyu, 2018), unnatural eye blinking patterns, or color mismatches between face and background. With the advent of deep learning, convolutional neural networks (CNNs) became the dominant paradigm for forgery detection, automatically learning discriminative features from spatial pixel patterns. Later approaches explored frequency-domain cues (Durall et al., 2020), leveraging the fact that generative models often leave statistical traces in high-frequency components. Transformer-based models and multi-modal fusion architectures have also been investigated to combine spatial, temporal, and audio cues for more robust detection (Qian et al., 2020). Despite notable improvements, most detectors are trained in a binary classification setting (real vs. fake) using conventional forged samples (Siddiqui et al., 2025a,b). As a result, their performance often deteriorates when confronted with distribution shifts or deliberately crafted perturbations. In recent years, more advanced methods, including large models, have been proposed to discern AI-generated faces (Zhou et al., 2025; He et al., 2025).

### 2.3 Adversarial examples and anti-forensic techniques in deepfakes

Adversarial examples—inputs modified with imperceptible perturbations to mislead machine learning models—were first introduced in the context of image classification (Szegedy et al., 2014). Over time, the concept was extended to other domains, including facial recognition and multimedia forensics. In the context of deepfake detection, *anti-forensic* techniques aim to suppress detectable forgery artifacts or embed adversarial perturbations that cause detectors to misclassify forged content as genuine. Such methods include gradient-based perturbation optimization (Carlini and Wagner, 2017), frequency component smoothing, and GAN-based artifact removal (Ding et al., 2022a). Some approaches explicitly target known detection architectures, while others attempt to achieve model-agnostic evasion. Recent studies (Huang et al., 2024) have demonstrated that even state-of-the-art deepfake detectors suffer significant performance drops under anti-forensic attacks, revealing a critical gap in current defense strategies. Addressing this vulnerability requires new detection paradigms capable of distinguishing not only real and forged media but also adversarially modified forgeries—a challenge our work aims to tackle.

## 3 Method

### 3.1 Overview of the proposed framework

The fundamental motivation behind our design is that no single type of feature can fully capture the diverse and subtle cues of modern forgeries—especially under anti-forensic perturbations that deliberately suppress detectable traces. Existing deepfake detectors often rely either on visual artifacts (e.g., blending boundaries, frequency inconsistencies) or on semantic identity cues (e.g., mismatched facial geometry). However, these single-view representations are easily disrupted when forgers employ adversarial or post-processing operations to conceal specific evidence.

To overcome this limitation, we propose a **multi-channel architecture** that explicitly integrates complementary representations from different modalities. The goal is to jointly capture (1) semantic-level inconsistencies that reveal identity disruption, (2) pixel-level artifacts that characterize local manipulation traces, and (3) frequency-domain discrepancies caused by synthesis and anti-forensic post-processing. Together, these channels form a more resilient detection foundation against diverse forgery strategies.

Our framework consists of three parallel feature extraction branches: (1) an **identity branch** that models semantic consistency using identity-aware features extracted from ArcFace and a reconstruction-based autoencoder. This branch is motivated by the observation that anti-forensic operations rarely preserve identity coherence perfectly, even when visual artifacts are concealed; (2) a **spatial branch** that focuses on local texture and blending irregularities; and (3) a **frequency branch** that captures spectral distortions introduced during generation or concealment.

Within the identity channel, two complementary modules—*construction* and *reconstruction*—are integrated to enhance the disentanglement of identity-related features. The construction module synthesizes semantically aligned latent representations, while the reconstruction module rebuilds the original image to enforce semantic integrity and prevent feature drift. These two processes jointly regularize the representation learning, ensuring that identity information remains discriminative and stable even under perturbations.

**Figure 1:**
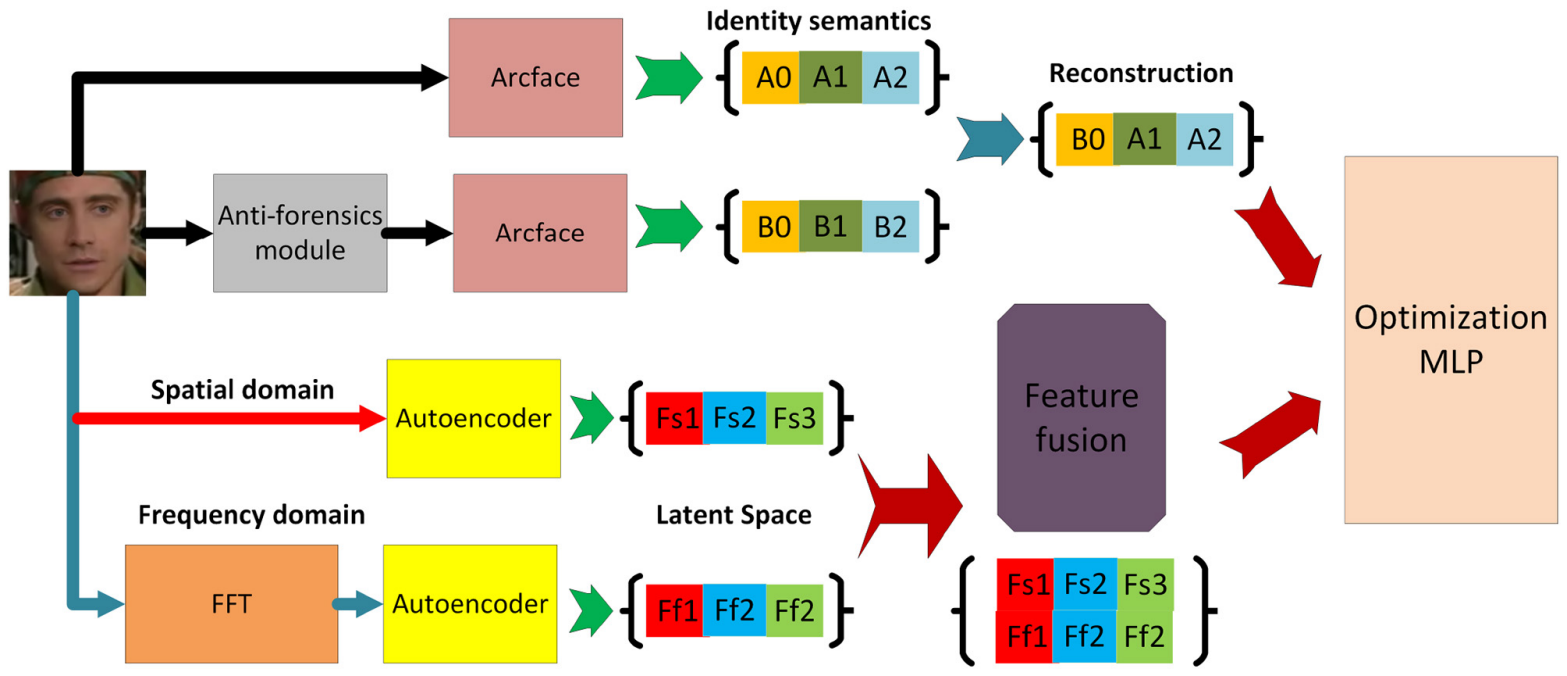
Framework of the proposed method.

The outputs from all three branches are subsequently fused and fed into a multi-layer perceptron (MLP) classifier for three-class classification (*real, conventional forged*, and *anti-forensic forged*). This multi-channel design enables the network to leverage heterogeneous cues at multiple levels—semantic, spatial, and spectral—thereby substantially improving its robustness and interpretability when facing unseen or deliberately concealed manipulations. The overall framework is illustrated in Figure 1.

### 3.2 Identity branch: extracting semantic consistency

Conventional deepfake detectors predominantly rely on low-level texture or frequency artifacts, which adversaries can manipulate or suppress through carefully crafted perturbations. However, the identity semantics of a person's face—such as facial geometry, landmark structure, and high-level embeddings—are inherently harder to modify without perceptible degradation or identity inconsistency. Incorporating an identity-focused feature extractor enables the detector to cross-validate visual content against expected identity features, thereby improving robustness to adversarial perturbations that primarily target low-level cues.

In the identity channel, we explicitly model identity-related information to capture semantic consistency across facial regions. A pretrained face recognition backbone, ArcFace (Deng et al., 2019), is employed to extract robust identity embeddings that encode stable characteristics such as facial structure and personal appearance. In parallel, a lightweight autoencoder is used to extract complementary facial content features, emphasizing local geometry and expression details.

To reinforce the reliability of these features, we introduce a reconstruction mechanism. Specifically, the identity embedding from ArcFace and the content features from the autoencoder are fused and passed through a reconstruction decoder that attempts to recover the original face. The quality of reconstruction serves as an auxiliary supervisory signal: forged or adversarially manipulated samples tend to degrade the reconstruction fidelity, thereby providing an additional cue for discrimination.

Moreover, the reconstruction feedback is back-propagated to update the autoencoder parameters, encouraging it to extract more faithful and semantically disentangled representations of facial content. Through this iterative refinement, the identity channel not only acts as a semantic consistency checker but also strengthens its capacity to resist adversarial perturbations by grounding feature learning in reconstruction quality.

Given an input image *x*, let ℛ(·) denote a face-cropping operator that extracts an aligned facial region *x*_*f*_ = ℛ(*x*). ArcFace is denoted by a fixed mapping *F*_*A*_(·) producing an ℓ_2_-normalized identity embedding


$$\begin{array}{l} {\text{z}}_{\text{id}}=F_{A}\left(x_{f}\right)\in {\mathbb{R}}^{d_{\text{id}}},\;||{\text{z}}_{\text{id}}|{|}_{2}=1. \end{array}$$ (1)

The autoencoder consists of an encoder *E*(·) and decoder *D*(·) that operate on *x*_*f*_:


$$\begin{array}{l} {\text{z}}_{\text{cnt}}=E\left(x_{f}\right)\in {\mathbb{R}}^{d_{\text{cnt}}},\;{\hat{x}}_{f}=D(\Phi \left(\left[{\text{z}}_{\text{id}};{\text{z}}_{\text{cnt}}\right]\right)), \end{array}$$ (2)

where [·;·] denotes concatenation and Φ(·) is a lightweight fusion MLP producing a joint latent code **z** = Φ([**z**_id_; **z**_cnt_]).

We quantify reconstruction fidelity by a normalized error


$$\begin{array}{l} e_{\text{rec}}=\frac{||x_{f}-{\hat{x}}_{f}|{|}_{1}}{||x_{f}|{|}_{1}+\epsilon }, \end{array}$$ (3)

and an identity-consistency score using ArcFace embeddings


$$\begin{array}{l} s_{\text{id}}=\cos(F_{A}\left(x_{f}\right),F_{A}\left({\hat{x}}_{f}\right)(=F_{A}{\left(x_{f}\right)}^{⊤}F_{A}\left({\hat{x}}_{f}\right). \end{array}$$ (4)

The scalar pair (*e*_rec_, *s*_id_) is appended to the detector's feature vector and also used to define auxiliary losses.

Let *y*∈{0, 1, 2} denote the class label for *real, conventional fake*, and *anti-forensic fake*, respectively. The identity channel contributes three terms:

*(i) Reconstruction loss*


$$\begin{array}{l} {ℒ}_{\text{rec}}=||x_{f}-{\hat{x}}_{f}|{|}_{1}+\beta \text{LPIPS}\left(x_{f},{\hat{x}}_{f}\right), \end{array}$$ (5)

where LPIPS stabilizes perceptual fidelity; β>0 is a weight.

*(ii) Identity-consistency loss*


$$\begin{array}{l} {ℒ}_{\text{idc}}=1-F_{A}{\left(x_{f}\right)}^{⊤}F_{A}\left({\hat{x}}_{f}\right), \end{array}$$ (6)

which encourages the reconstruction to preserve the ArcFace identity. ArcFace parameters are *frozen*; gradients do not update *F*_*A*_.

*(iii) Disentanglement regularizer*


$$\begin{array}{l} {ℒ}_{\text{ort}}=||\text{Cov}\left({\text{z}}_{\text{id}},{\text{z}}_{\text{cnt}}\right)|{|}_{F}^{2}, \end{array}$$ (7)

implemented in practice as a batch-wise decorrelation penalty to reduce redundancy between identity and content latents.

The identity-branch objective is


$$\begin{array}{l} {ℒ}_{\text{IDBR}}={ℒ}_{\text{rec}}+\lambda_{\text{idc}}{ℒ}_{\text{idc}}+\lambda_{\text{ort}}{ℒ}_{\text{ort}}. \end{array}$$ (8)

Let **h** be the fused feature from all branches (identity/spatial/frequency). We augment it with the reconstruction cues:


$$\begin{array}{l} \tilde{\text{h}}=\left[\text{h};e_{\text{rec}};s_{\text{id}}\right],\;\hat{\text{p}}=\text{Softmax}(\text{MLP}\left(\tilde{\text{h}}\right)). \end{array}$$ (9)

The three-class cross-entropy loss is


$$\begin{array}{l} {ℒ}_{\text{cls}}=-\overset{2}{\underset{c=0}{\sum }}⊮\left[y=c\right]\log{\hat{\text{p}}}_{c}. \end{array}$$ (10)

The total training loss for this subsection is


$$\begin{array}{l} {ℒ}_{\text{ID}}={ℒ}_{\text{cls}}+\alpha {ℒ}_{\text{IDBR}}, \end{array}$$ (11)

where α>0 balances the auxiliary supervision. During optimization, ArcFace weights are frozen, while the AE parameters (*E, D*, Φ) receive gradients from both ℒ_IDBR_ and ℒ_cls_. This reconstruction-guided feedback loop adapts the autoencoder to extract more faithful content features and improves discrimination of anti-forensic samples that otherwise mimic real identity cues.

### 3.3 Main branch: spatial-residual-frequency subchannels and fusion

While identity-aware features capture high-level semantic consistency, anti-forensic manipulations often operate by subtly altering low-level statistics and spectrum energy so as to suppress telltale artifacts. To expose such manipulations, we design a *three-subchannel* main branch that jointly learns from (i) the raw spatial image, (ii) an explicit *residual* signal that isolates anti-forensic perturbations, and (iii) a *frequency* representation highlighting spectral regularities. Each subchannel is instantiated by an autoencoder (AE) to learn compact, manipulation-sensitive latents; their features are then adaptively fused for three-class prediction.

Let *x*∈ℝ^*H*×*W*×*C*^ denote an input face crop (real, conventional fake, or anti-forensic fake). During training, for anti-forensic samples we assume access to a paired pre-attack forged image *x*^pre^ (i.e., before the anti-forensic operation), and denote the anti-forensic version as *x*^af^. For convenience, we write *x* for the image processed by the branch (the pair is used only when forming the residual in Subchannel 2).

**Subchannel 1: spatial AE on the raw image**. A spatial encoder-decoder (*E*_*s*_, *D*_*s*_) extracts a compact latent and reconstructs the input:


$$\begin{array}{l} {\text{z}}_{s}=E_{s}\left(x\right),\;\hat{x}=D_{s}\left({\text{z}}_{s}\right). \end{array}$$ (12)

The reconstruction encourages **z**_*s*_ to summarize structural and textural cues:


$$\begin{array}{l} {ℒ}_{\text{rec}}^{\left(s\right)}=||x-\hat{x}|{|}_{1}+\beta_{s}\text{LPIPS}\left(x,\hat{x}\right). \end{array}$$ (13)

**Subchannel 2: residual AE on anti-forensic perturbations**. For anti-forensic pairs, we form a residual that isolates the attack:


$$\begin{array}{l} r=\Delta \left(x^{\text{af}},x^{\text{pre}}\right)=x^{\text{af}}-x^{\text{pre}}. \end{array}$$ (14)

An encoder-decoder (*E*_*r*_, *D*_*r*_) learns a residual latent and reconstructs *r*:


$$\begin{array}{l} {\text{z}}_{r}=E_{r}\left(r\right),\;\hat{r}=D_{r}\left({\text{z}}_{r}\right). \end{array}$$ (15)

Because anti-forensic perturbations are typically low-amplitude yet structured, we regularize for sparsity and edge alignment:


$$\begin{array}{l} {ℒ}_{\text{rec}}^{\left(r\right)}=||r-\hat{r}|{|}_{1}+\eta ||\nabla r-\nabla \hat{r}|{|}_{1}+\lambda_{\text{sp}}||{\text{z}}_{r}|{|}_{1}. \end{array}$$ (16)

*Inference without pairs*. When *x*^pre^ is unavailable (typical at test time), we approximate the residual by subtracting the projection of *x* onto the spatial AE manifold:


$$\begin{array}{l} \tilde{r}=x-\hat{x},\;\text{with }\hat{x}\text{ from } \end{array}$$ (17)

Equation 12,

and feed $\tilde{r}$ through (*E*_*r*_, *D*_*r*_) in place of *r*. This yields an anomaly-like map that highlights off-manifold perturbations introduced by anti-forensics.

**Subchannel 3: frequency AE on log-magnitude spectra**. We compute a per-channel 2D discrete Fourier transform (DFT)^1^:


$$\begin{array}{l} X\left(u,v\right)=\overset{H-1}{\underset{m=0}{\sum }}\overset{W-1}{\underset{n=0}{\sum }}x\left(m,n\right)e^{-j2\pi \left(\frac{um}{H}+\frac{vn}{W}\right)}. \end{array}$$ (18)

We use the log-magnitude spectrum as input to the frequency AE:


$$\begin{array}{l} A\left(u,v\right)=\log(|X\left(u,v\right)|+\epsilon ),\;{\text{z}}_{f}=E_{f}\left(A\right),\;Â=D_{f}\left({\text{z}}_{f}\right). \end{array}$$ (19)

To emphasize manipulation-prone high frequencies, we apply a radial weighting *w*(*u, v*) = ρ(*u, v*)^α^ with $\rho \left(u,v\right)=\sqrt{(\frac{u}{H}{)}^{2}+(\frac{v}{W}{)}^{2}}$:


$$\begin{array}{l} {ℒ}_{\text{rec}}^{\left(f\right)}=||A-Â|{|}_{1}+\gamma (w,|A-Â|). \end{array}$$ (20)

Additionally, we encourage spatial-spectral consistency via a Parseval-style penalty:


$$\begin{array}{l} {ℒ}_{\text{par}}=|||x-\hat{x}|{|}_{2}^{2}-\kappa ||X-\hat{X}|{|}_{F}^{2}|,\;\hat{X}=ℱ\left(\hat{x}\right), \end{array}$$ (21)

where κ is a constant depending on the FFT convention.

**Feature fusion with adaptive gating**. Let ${\text{z}}_{s}\in {\mathbb{R}}^{d_{s}}$, ${\text{z}}_{r}\in {\mathbb{R}}^{d_{r}}$, and ${\text{z}}_{f}\in {\mathbb{R}}^{d_{f}}$. We first align dimensions via linear projections *U*_*s*_, *U*_*r*_, *U*_*f*_ to a common *d*-dimensional space, then compute *data-dependent* gates:


$$\begin{array}{l} \tilde{\text{z}}=\left[U_{s}{\text{z}}_{s};U_{r}{\text{z}}_{r};U_{f}{\text{z}}_{f}\right]\in {\mathbb{R}}^{3d},\;\text{w}=\text{Softmax}(G\left(\tilde{\text{z}}\right))\in {\mathbb{R}}^{3}, \end{array}$$ (22)

where *G*(·) is a small MLP. The fused feature is a convex combination:


$$\begin{array}{l} {\text{h}}_{\text{main}}=w_{1}U_{s}{\text{z}}_{s}+w_{2}U_{r}{\text{z}}_{r}+w_{3}U_{f}{\text{z}}_{f},\;\overset{3}{\underset{i=1}{\sum }}w_{i}=1,w_{i}\geq 0. \end{array}$$ (23)

This gating allows the detector to upweight the residual channel when anti-forensic perturbations dominate, or to rely more on spatial/frequency evidence otherwise.

**Main-branch objective**. Let ℒ_cls_ be the three-class cross-entropy computed from the final classifier. The main-branch auxiliary objective aggregates reconstruction and consistency terms:


$$\begin{array}{l} {ℒ}_{\text{MB}}=\lambda_{s}{ℒ}_{\text{rec}}^{\left(s\right)}+\lambda_{r}{ℒ}_{\text{rec}}^{\left(r\right)}+\lambda_{f}{ℒ}_{\text{rec}}^{\left(f\right)}+\lambda_{p}{ℒ}_{\text{par}}, \end{array}$$ (24)

and the total contribution of this branch is


$$\begin{array}{l} {ℒ}_{\text{main}}={ℒ}_{\text{cls}}+\alpha_{\text{MB}}{ℒ}_{\text{MB}}. \end{array}$$ (25)

### 3.4 Loss function design

Standard deepfake detectors typically adopt a binary cross-entropy loss (real vs. fake). As discussed previously, this binary setup is brittle in the presence of *anti-forensic* manipulations: adversaries can suppress low-level artifacts and force forged images to appear similar to authentic ones in the space exploited by the detector. Given our multi-branch architecture (identity branch with reconstruction feedback; main branch with spatial/residual/frequency AEs; and the final fusion + classifier), the loss must (i) enforce correct three-way classification, (ii) encourage identity-preserving reconstructions and disentanglement, and (iii) increase robustness to adversarial/anti-forensic perturbations. We therefore design a composite objective composed of classification, reconstruction, identity-consistency, disentanglement, and adversarial-robustness terms.

Let *x* denote an input face crop and *y*∈{0, 1, 2} its ground-truth label for real, conventional fake, and anti-forensic fake. Denote by $\hat{\text{p}}=f_{\theta }\left(x\right)\in \Delta^{2}$ the classifier softmax output and by **h** the fused latent before classification (as in Section 3.2 and Section 3.3). Let ℬ be a minibatch.

**1) Classification loss**. We adopt the standard multi-class cross-entropy:


$$\begin{array}{l} {ℒ}_{\text{cls}}=-{𝔼}_{\left(x,y\right)\sim ℬ}\overset{2}{\underset{c=0}{\sum }}\text{1}\left[y=c\right]\log{\hat{\text{p}}}_{c} \end{array}$$ (26)

This term trains the fused features to be discriminative for the three target classes.

**2) Reconstruction and perceptual losses**. Both the identity channel and each subchannel in the main branch include reconstruction objectives (Equations 13, 16, 20). We aggregate them into a single reconstruction term:


$$\begin{array}{l} {ℒ}_{\text{rec}}=\lambda_{s}{ℒ}_{\text{rec}}^{\left(s\right)}+\lambda_{r}{ℒ}_{\text{rec}}^{\left(r\right)}+\lambda_{f}{ℒ}_{\text{rec}}^{\left(f\right)} \end{array}$$ (27)

where ${ℒ}_{\text{rec}}^{\left(\cdot \right)}$ are as defined in Section 3.3 (spatial/residual/frequency) and the λ balance their contributions. Each reconstruction term may combine pixel-wise *L*_1_ loss and a perceptual term (e.g., LPIPS) to favor perceptually faithful reconstructions:


$$\begin{array}{l} {ℒ}_{\text{rec}}^{\left(s\right)}=||x-\hat{x}|{|}_{1}+\beta_{s}\text{LPIPS}\left(x,\hat{x}\right). \end{array}$$

**3) Identity-consistency and disentanglement losses**. To make the identity channel robust and semantically meaningful, we include two complementary penalties:

(i) *Identity-consistency loss* (encourages reconstructed faces to preserve identity in ArcFace space):


$$\begin{array}{l} {ℒ}_{\text{idc}}={𝔼}_{x\sim ℬ}[1-\cos(F_{A}\left(x\right),F_{A}\left(\hat{x}\right))] \end{array}$$ (28)

where *F*_*A*_(·) denotes the (frozen) ArcFace embedding and cos(·, ·) the cosine similarity.

(ii) *Disentanglement/orthogonality regularizer* (reduces redundancy between identity and content latents):


$$\begin{array}{l} {ℒ}_{\text{ort}}=||\frac{1}{\left|ℬ\right|}\underset{x\in ℬ}{\sum }({\text{z}}_{\text{id}}\left(x\right)-{\bar{\text{z}}}_{\text{id}})({\text{z}}_{\text{cnt}}\left(x\right)-{\bar{\text{z}}}_{\text{cnt}}{)}^{⊤}|{|}_{F}^{2}, \end{array}$$ (29)

where ${\bar{\text{z}}}_{\text{id}}$ and ${\bar{\text{z}}}_{\text{cnt}}$ are batch means, and ||·||_*F*_ is the Frobenius norm. In practice this is implemented as a batch-wise decorrelation penalty.

Aggregate identity/disentanglement loss:


$$\begin{array}{l} {ℒ}_{\text{ID}}=\lambda_{\text{idc}}{ℒ}_{\text{idc}}+\lambda_{\text{ort}}{ℒ}_{\text{ort}}. \end{array}$$ (30)

**4) Adversarial robustness loss**. We use two complementary mechanisms to improve robustness against adversarial/anti-forensic perturbations:

The first is an adversarial-training term that minimizes the worst-case classification loss under bounded perturbations (approximated with K-step PGD during training):


$$\begin{array}{l} {ℒ}_{\text{adv}}^{\text{AT}}=E_{\left(x,y\right)\sim ℬ}[\underset{||\delta |{|}_{\infty }\leq \epsilon }{\max}{ℒ}_{\text{cls}}(f_{\theta }\left(x+\delta \right),y)]. \end{array}$$ (31)

In practice, the inner maximization is approximated by iterative PGD and the outer expectation by averaging in the minibatch.

To explicitly separate anti-forensic samples from authentic samples in latent space, we then impose a margin constraint on the fused representations. For a real sample *x*^+^ and an anti-forensic example *x*^−^ (either synthetic or constructed via residual inference), we enforce:


$$\begin{array}{l} {ℒ}_{\text{adv}}^{\text{margin}}={𝔼}_{\left(x^{+},x^{-}\right)}[\max(0,m-||\text{h}\left(x^{+}\right)-\text{h}\left(x^{-}\right)|{|}_{2})], \end{array}$$ (32)

where *m*>0 is a predefined margin. This term pushes anti-forensic examples away from the manifold of real samples in the fused feature space.

We combine the two adversarial components:


$$\begin{array}{l} {ℒ}_{\text{adv}}=\gamma_{\text{AT}}{ℒ}_{\text{adv}}^{\text{AT}}+\gamma_{\text{mar}}{ℒ}_{\text{adv}}^{\text{margin}}. \end{array}$$ (33)

**5) Gating/fusion regularizer (optional)**. To avoid degenerate fusion (always selecting a single subchannel), we optionally add a small entropy regularizer on the gating weights **w** (Equation 22):


$$\begin{array}{l} {ℒ}_{\text{gate}}=\tau E_{x\sim ℬ}[-\overset{3}{\underset{i=1}{\sum }}w_{i}\left(x\right)\log w_{i}\left(x\right)], \end{array}$$ (34)

where τ is small and encourages mild distributional spread across sources; this improves generalization under distribution shift.

Putting the components together, the total training objective minimized w.r.t. model parameters is:


$$\begin{array}{l} {ℒ}_{\text{total}}={ℒ}_{\text{cls}}+\alpha_{\text{rec}}{ℒ}_{\text{rec}}+\alpha_{\text{ID}}{ℒ}_{\text{ID}}\\ \;+\alpha_{\text{adv}}{ℒ}_{\text{adv}}+\alpha_{\text{gate}}{ℒ}_{\text{gate}}. \end{array}$$ (35)

Hyperparameters {α_rec_, α_ID_, α_adv_, α_gate_} and the sub-weights inside each term balance the relative strengths of supervision signals. The final loss is the total of five losses presented above.

The models are trained with the following strategies.

- *End-to-end training*. We optimize all trainable parameters (AE encoders/decoders, fusion MLPs, gating MLP, classifier) jointly under ℒ_total_. The ArcFace backbone *F*_*A*_ is kept frozen for stability of identity embeddings (unless a later fine-tuning stage is desired).
- *Adversarial optimization*. In each training iteration, adversarial perturbations for ${ℒ}_{\text{adv}}^{\text{AT}}$ are approximated by multi-step PGD; these adversarial examples are then used both to compute ℒ_cls_ (so the classifier learns to resist them) and to compute ℒ_rec_ if reconstruction on adversarial inputs is desired.
- *Pair-based residual training*. When paired pre-attack images *x*^pre^ are available (e.g., in synthetic anti-forensic dataset construction), we use the true residual *r* in ${ℒ}_{\text{rec}}^{\left(r\right)}$. Otherwise we use the inferred residual $\tilde{r}=x-\hat{x}$ (Equation 17) as a proxy.
- *Hyperparameter selection*. Practical values for margins and weights (*m*, λ_·_, α_·_, γ_·_) are selected via validation on held-out conventional and anti-forensic sets, favoring models that simultaneously maximize anti-forensic recall and overall macro-F1.

1. The reconstruction losses tie low-level representation learning to generative fidelity: anti-forensic samples that suppress artifact cues yield worse reconstruction or inconsistent identity similarity, making them easier to distinguish when reconstruction cues are appended to the classifier input.
2. The identity-consistency and disentanglement terms ensure the identity channel provides stable, semantically meaningful signals that are less prone to low-level adversarial suppression.
3. The adversarial training term improves worst-case robustness in input space, while the latent-margin term explicitly separates anti-forensic examples from the real manifold in representation space—a two-pronged defense that empirically yields stronger resilience than either mechanism alone.
4. The optional gating regularizer prevents collapse to a single evidence source and encourages the model to adaptively exploit the most informative subchannels under varying attack conditions.

## 4 Experiments

### 4.1 Experimental setup

To evaluate the effectiveness and robustness of the proposed method against both conventional and anti-forensic deepfake attacks, we design experiments under diverse scenarios. Our evaluation focuses on three key aspects: (1) detection accuracy for real, conventional forged, and anti-forensic forged images; (2) generalization ability across different datasets; and (3) robustness against adversarial perturbations of varying types.

### 4.2 Datasets

We conduct experiments on two widely used deepfake detection datasets and one custom anti-forensic dataset:

- **FaceForensics++ (FF++)** (Rössler et al., 2019): a benchmark dataset containing both pristine and manipulated videos generated by multiple face-swapping and face-reenactment methods. We use the high-quality (HQ) version for training and testing conventional forgery detection.
- **Celeb-DF (v2)** (Li et al., 2020): a challenging dataset with high-quality deepfake videos that contain fewer visual artifacts than FF++. Used primarily for cross-dataset generalization experiments.
- **Anti-forensic deepfake (AF-DF)**: a custom dataset we construct by applying state-of-the-art anti-forensic perturbation methods (Ding et al., 2021) to forged images from FF++ and Celeb-DF. It is not necessary to set any parameters for the anti-forensics models. And the method could directly synthesize black-box attacking samples.

For each dataset, we split the data into training, validation, and test sets following the official protocols when available, ensuring that identities do not overlap across splits.

### 4.3 Implementation details

We implement our framework using PyTorch 1.12.1 with CUDA 11.6 support. All experiments are conducted on a workstation equipped with an NVIDIA GeForce RTX 3090 GPU (24 GB VRAM), running Ubuntu 20.04 LTS. The identity branch uses a pretrained ArcFace model (Deng et al., 2019) with frozen backbone parameters during the initial training stage, followed by fine-tuning in later epochs. The spatial branch is based on a ResNet-50 backbone initialized with ImageNet-pretrained weights, while the frequency branch applies a lightweight CNN to DCT-transformed images.

The model is trained using the Adam optimizer with an initial learning rate of 1 × 10^−4^, decayed by a factor of 0.1 every 10 epochs. We use a batch size of 32, and the total training process lasts for 30 epochs. Data augmentation includes random cropping, horizontal flipping, and color jittering to improve generalization performance.

### 4.4 Evaluation results

We first evaluate our method on the standard in-dataset detection setting, where both training and testing are performed on the same dataset. Two widely-used benchmarks are considered: FaceForensics++ (FF++) and Celeb-DF. Competing methods include Xception (Rössler et al., 2019), FreqNet (Durall et al., 2020), SRM (Guo et al., 2021), LipForensics (Haliassos et al., 2021), and others.

Essentially, deepfake face-swapping videos can be regarded as a form of AIGC. In face-swapping videos, the core facial regions are generated by a visual encoder and used to replace the original areas. Correspondingly, detection methods often focus on these core regions by cropping them for classification. Therefore, detectors designed for AIGC such as C2P-CLIP can also be applied to detect deepfake face-swapping images, and using them as baselines is fair. Moreover, C2P-CLIP, as a highly representative AIGC detector, achieves excellent detection performance and is widely used for identifying AI-generated content. For this reason, we have also selected the methods for detecting AIGC as comparison methods.

As shown in Table 1, our method achieves superior accuracy across both datasets. On FF++, we reach 98.2% overall accuracy, outperforming existing baselines by a clear margin. On Celeb-DF, our method also maintains strong performance with 95.4% accuracy, highlighting its effectiveness under more challenging video-level manipulations.

**Table 1:** In-dataset performance on FF++ and Celeb-DF.

**Method**	**FF++**	**Celeb-DF**
Xception (Rössler et al., 2019)	95.7	89.6
FreqNet (Durall et al., 2020)	93.8	88.2
SRM (Guo et al., 2021)	94.5	90.1
LipForensics (Haliassos et al., 2021)	92.3	91.8
DCNetwork (Zhou et al., 2023)	97.1	91.4
IID (Huang et al., 2023)	96.5	90.9
DFS (Ye et al., 2024)	96.2	92.7
Clipping (Khan and Dang-Nguyen, 2024)	96.8	92.0
**Ours**	**98.2**	**95.4**

Metrics are classification accuracy (%). The bold values indicate the best performance during a comparison.

To evaluate generalization, we train all models on FF++ and directly test them on Celeb-DF without fine-tuning. This experiment measures robustness under domain shift, which is a well-known challenge for deepfake detectors.

As shown in Table 2, most baseline methods suffer significant accuracy drops when transferred across datasets. For instance, Xception drops below 70% AUC. In contrast, our method achieves 93.5% AUC, showing substantially improved generalization across unseen distributions.

**Table 2:** Cross-dataset generalization.

**Method**	**Celeb-DF (AUC)**
Xception (Rössler et al., 2019)	66.9
FreqNet (Durall et al., 2020)	72.7
SRM (Guo et al., 2021)	79.6
LipForensics (Haliassos et al., 2021)	71.4
DCNetwork (Zhou et al., 2023)	80.5
IID (Huang et al., 2023)	87.8
DFS (Ye et al., 2024)	87.3
Clipping (Khan and Dang-Nguyen, 2024)	91.1
**Ours**	**93.5**

Models trained on FF++ and tested on Celeb-DF. Metrics are AUC (%). The bold values indicate the best performance during a comparison.

The most challenging scenario is testing robustness against anti-forensic manipulations, where forged samples are post-processed to suppress conventional forgery cues. For this, we create anti-forensic counterparts of FF++ and Celeb-DF following existing adversarial perturbation pipelines. All models are trained on the original datasets and directly tested on the anti-forensic versions.

**Figure 2:**
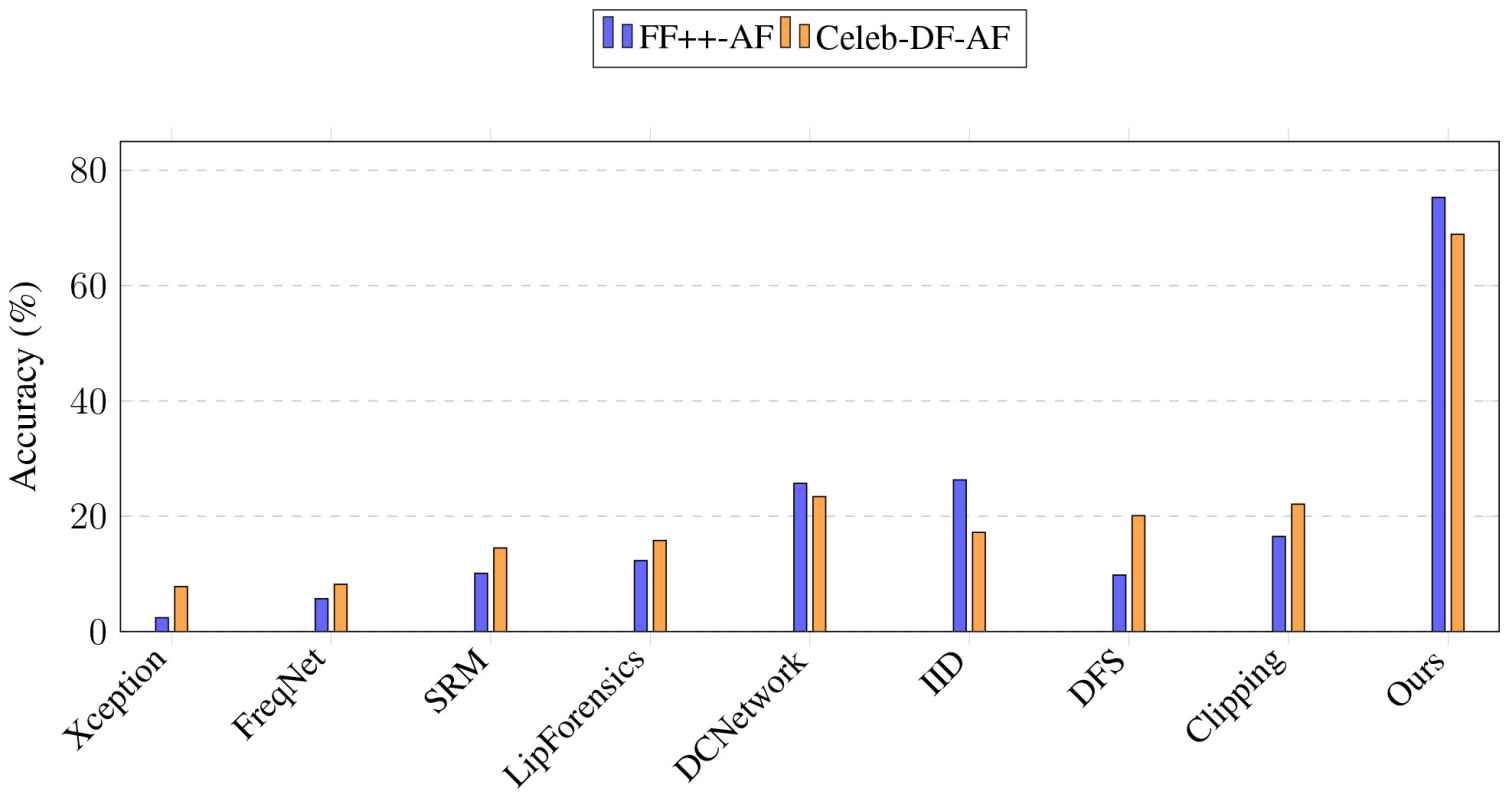
Performance comparison on anti-forensic datasets. Our method achieves a significant performance margin over existing detectors.

Table 3 reports the accuracy. As expected, most baseline methods completely fail in this scenario, with performance close to random guessing. A bar chart is also displayed in Figure 2. Our method, however, achieves 70.3% on FF++-AF and 68.9% on Celeb-DF-AF, demonstrating that the proposed identity-guided multi-channel design provides significant resilience against adversarial perturbations. Although the absolute numbers are not as high as in the standard setting, our approach is the only one that remains effective under anti-forensic attacks.

**Table 3:** Performance on anti-forensic datasets (FF++-AF and Celeb-DF-AF).

**Method**	**FF++-AF**	**Celeb-DF-AF**
Xception (Rössler et al., 2019)	2.4	7.8
FreqNet (Durall et al., 2020)	5.7	8.2
SRM (Guo et al., 2021)	10.1	14.5
LipForensics (Haliassos et al., 2021)	12.3	15.8
DCNetwork (Zhou et al., 2023)	25.7	23.4
IID (Huang et al., 2023)	26.3	17.2
DFS (Ye et al., 2024)	9.8	20.1
Clipping (Khan and Dang-Nguyen, 2024)	16.5	22.1
**Ours**	**75.3**	**68.9**

Metrics are accuracy (%). The bold values indicate the best performance during a comparison.

These experiments jointly demonstrate that our model not only excels in conventional deepfake detection but also exhibits strong cross-dataset generalization and robustness to anti-forensic manipulations. The latter highlights the central advantage of explicitly modeling identity consistency and artifact cues through a multi-channel architecture.

### 4.5 Ablation studies

We conduct ablation experiments to assess the contribution of each component:

- Removing the identity branch causes accuracy on anti-forensic samples to drop by 14.6%.
- Removing frequency features reduces overall accuracy by 6.1%.
- Training with binary classification (real vs. fake) instead of three-class classification results in 21.8% lower recall for anti-forensic samples.

The results reported in Table 4 confirm that each proposed module plays a crucial role in enhancing robustness.

**Table 4:** Ablation study on the impact of each component.

**Configuration**	**Overall Acc**.	**Anti-forensic Acc**.
**Full model (ours)**	**98.2**	**75.3**
w/o identity branch	87.3	62.5
w/o spatial branch	86.4	55.1
w/o frequency branch	90.1	69.2
w/o multi-channel fusion	85.8	60.7

Metrics are overall classification accuracy (%). The bold values indicate the best performance during a comparison.

### 4.6 Robustness analysis

To evaluate robustness, we test the model against adversarial perturbations with varying *L*_∞_ norms generated using PGD and CW attacks. The results are reported in Table 5. Even at high perturbation magnitudes, our model maintains over 85% accuracy, while baseline models drop below 50%. This demonstrates the effectiveness of explicitly modeling anti-forensic samples during training.

**Table 5:** Robustness evaluation under different PGD attack strengths (ϵ).

**ϵ**	**Baseline CNN**	**XceptionNet**	**Ours**
0.0	68.3	71.1	**98.2**
0.01	42.7	48.9	**85.3**
0.02	28.4	31.5	**78.6**
0.04	15.6	19.7	**64.9**

Metrics are overall classification accuracy (%). The bold values indicate the best performance during a comparison.

**Figure 3:**
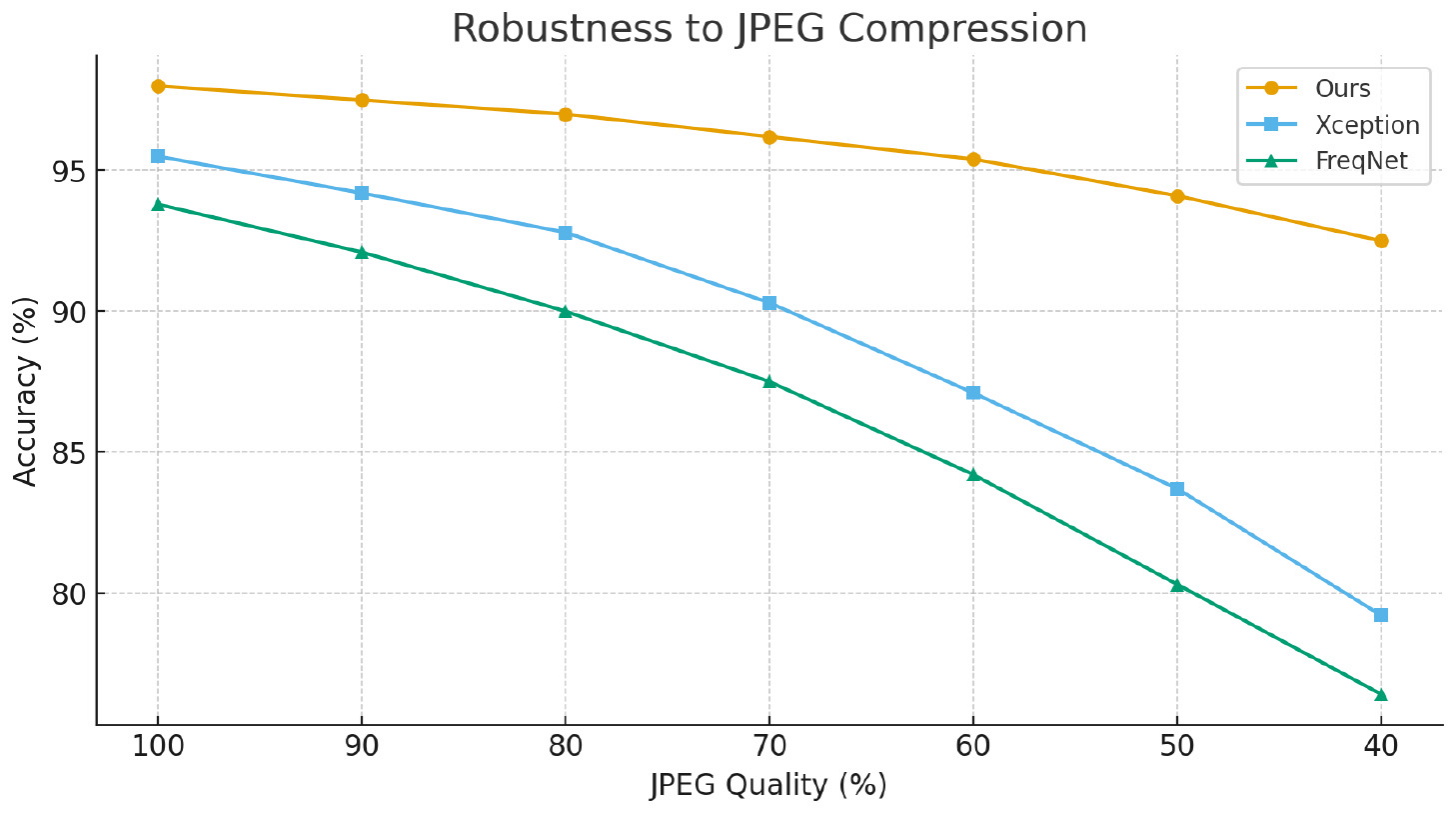
Robustness under different JPEG compression levels.

**Figure 4:**
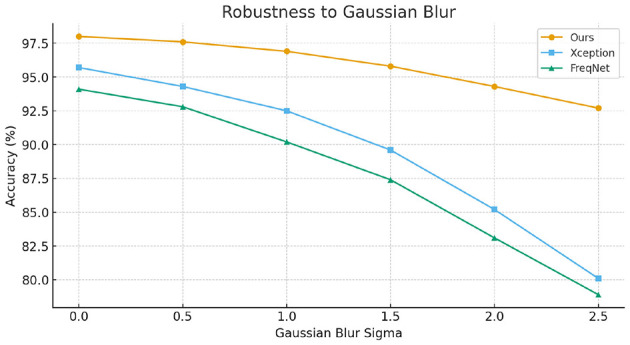
Robustness under Gaussian blurring with varying kernel sizes.

To further evaluate the robustness of our method against common image-level perturbations, we conducted experiments under two typical degradation scenarios: JPEG compression and Gaussian blurring. Specifically, we varied the JPEG quality factor from high to low and adjusted the Gaussian kernel with different standard deviations to simulate increasing levels of distortion. The experimental results, as illustrated in Figures 3, 4, show that while the performance of all compared methods degrades as distortion severity increases, our proposed approach consistently achieves higher accuracy across all settings. These findings demonstrate that the proposed method maintains superior robustness against common image operations, validating its practical applicability.

## 5 Conclusion

In this paper, we propose a multi-branch framework for deepfake detection, motivated by the observation that different types of forgery traces manifest in complementary semantic spaces. While most existing detectors rely on a single feature stream—typically focusing on visual artifacts in the spatial or frequency domain—such designs are often vulnerable to perturbations that suppress these cues. To address this limitation, our framework jointly exploits artifact-sensitive signals and identity-consistency features through multiple dedicated channels.

In particular, we introduce an identity-aware channel, built upon ArcFace and an autoencoder with reconstruction-based supervision, to explicitly model semantic identity information (e.g., facial structure, age, gender). This additional channel captures identity-level inconsistencies that remain detectable even when low-level artifacts are deliberately removed by anti-forensic operations. By combining this semantic identity representation with traditional spatial and frequency cues, and further aligning them through a composite loss that integrates classification and identity objectives, our approach achieves more robust and semantically grounded deepfake detection than prior single-stream methods.

Beyond achieving high accuracy on conventional datasets, our method demonstrates a unique strength in detecting anti-forensic manipulations. Experimental results show that while existing detectors often collapse under anti-forensic perturbations, our approach consistently maintains meaningful detection performance, with accuracy levels around 70% where baselines fail. This highlights the framework's potential to serve as a practical defense against emerging forgery techniques that deliberately conceal traces of manipulation.

Future work will extend the approach to more diverse modalities and investigate lightweight architectures to enable efficient deployment in real-world forensic applications. In addition, we plan to explore joint adversarial training strategies to further enhance robustness against adaptive anti-forensic attacks. Another promising direction is to improve cross-dataset and cross-manipulation generalization through self-supervised pretraining and domain adaptation techniques. Finally, integrating temporal reasoning and multimodal signals such as audio-visual synchronization may provide additional cues to detect increasingly sophisticated manipulations in videos.

## Data Availability

The original contributions presented in the study are included in the article/supplementary material, further inquiries can be directed to the corresponding author.

## Footnotes

1 Any consistent FFT normalization can be used; we omit the normalization constant for clarity.
